# Current challenges in prostate cancer: an interview with Prostate Cancer UK

**DOI:** 10.1186/s12916-015-0411-7

**Published:** 2015-07-24

**Authors:** Iain Frame, Sarah Cant

**Affiliations:** Prostate Cancer UK, 4th Floor, Counting House, 53 Tooley Street, London, SE1 2QN UK

**Keywords:** Diagnosis, Policy, Prostate cancer, Risk, Treatment

## Abstract

In this video Q&A, we talk to Iain Frame and Sarah Cant from Prostate Cancer UK about the current challenges in prostate cancer research and policy and how these are being addressed.

**Electronic supplementary material:**

The online version of this article (doi:10.1186/s12916-015-0411-7) contains supplementary material, which is available to authorized users.

## Introduction

**Fig. 1:**
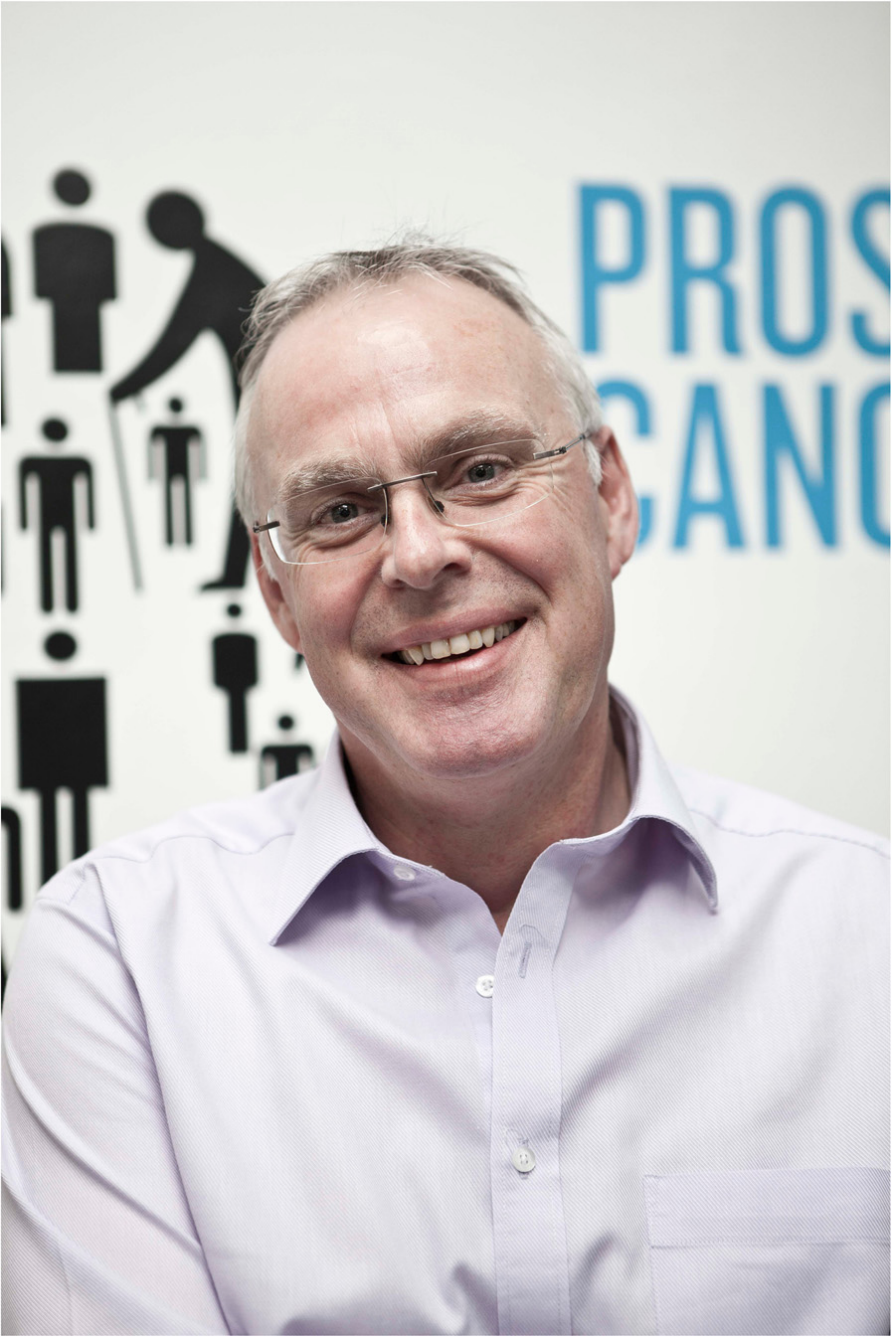
Dr. Iain Frame

Dr Iain Frame (Fig. 1) is Prostate Cancer UK’s Director of Research, responsible for overseeing the development and implementation of the charity’s research strategy. He previously worked as Research Director at Diabetes UK and in research management at the Wellcome Trust. Before that, he worked as a parasitologist and researcher exploring various aspects of molecular biology of a number of different parasites.

**Fig. 2:**
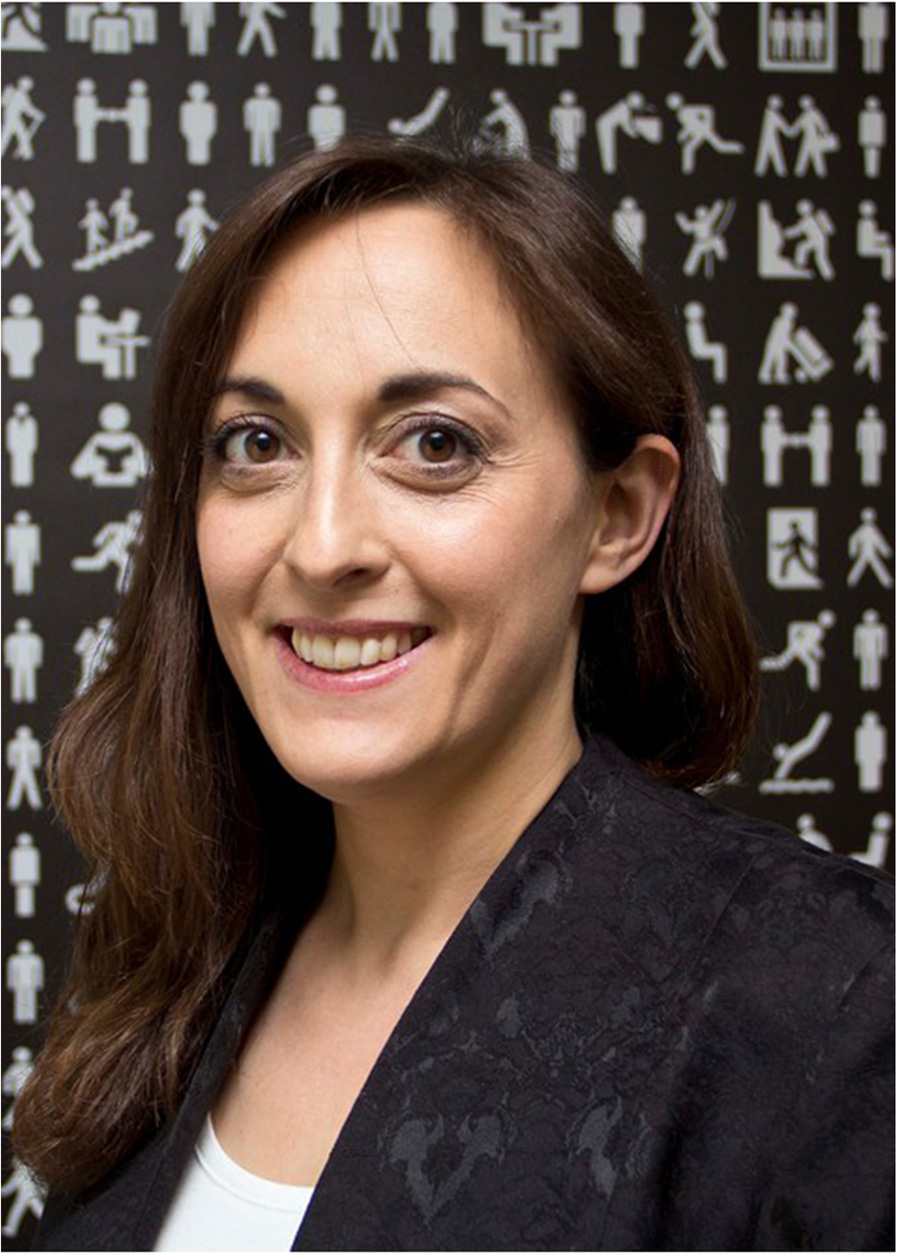
Dr. Sarah Cant

Dr Sarah Cant (Fig. 2) is Prostate Cancer UK’s Director of Policy and Strategy. She leads the charity’s work to determine what good quality treatments and care look like for men with prostate cancer, and influencing activities to ensure that men get the best possible care wherever they live.

Sarah was previously Head of Policy & Campaigns at Prostate Cancer UK, during which time she led a successful campaign to make abiraterone available across the UK. Sarah has a PhD in cell biology, and before joining Prostate Cancer UK, she worked at Breakthrough Breast Cancer and also completed a fellowship at the Parliamentary Office of Science & Technology (Video Q&A: Additional file 1).

## Full interview: edited transcript

### 1. What are the current challenges in prostate cancer research and policy, and why is it important to address them?

Iain Frame (IF): I think that there are three current challenges in prostate cancer research. The first is risk assessment – assessing a man’s risk of developing prostate cancer during his lifetime. The second is differential diagnosis and prognosis of aggressive versus non-aggressive cancer. The third is developing new treatments for advanced disease, but also working out how best to use existing treatments for optimal effect. These three areas are important because they are the three key areas that affect the outcome of a man’s diagnosis of having prostate cancer.

Sarah Cant (SC): I think there are quite a few challenges across prostate cancer, both for men with the disease and for the clinicians who are treating them. If we go from the start of the patient journey, the first issue is whether a man should have a test for prostate cancer. As I’m sure most people know, the prostate-specific antigen (PSA) test is not very accurate; it indicates that there is a problem with the prostate, it does not determine whether cancer is present. PSA testing leads to a number of other diagnostic techniques that can say whether a man has cancer, but not necessarily how aggressive it will be, and may lead to a lot of men having treatment that they may not have needed, for a cancer that may not have harmed them.

So men are faced with the decision of whether or not to have an inaccurate test in the first place. In fact, a lot of men don’t even know that they may be at risk for prostate cancer. There is quite low awareness of the disease amongst certain groups. For example, men from a black Afro-Caribbean ethnicity or with a family history of the disease may be at high risk. That is the first step: understanding whether or not a man is at high risk of the disease, whether or not he should have the test, and then what the implications are of being diagnosed with prostate cancer.

Then there are questions such as, should prostate cancer be treated? What treatment should a man have; what should he choose? What are the side effects? What are the impacts of particular treatments?

The next question is whether that treatment – and the support for any associated side effects – is actually available to a particular patient where he lives.

### 2. If we take a patient-centric view, what are the challenges men face at the moment when they deal with prostate cancer from diagnosis to treatment, and how can that situation be improved?

IF: The patient’s view informs everything that we do here at Prostate Cancer UK in terms of research. We have patients involved in research decision-making processes, so we hear from men about what’s important to them. The idea of a simple and effective risk assessment tool is something that’s high on the list. Another priority is being able to tell at the point of diagnosis whether a particular cancer is likely to be aggressive and kill you, or whether it will never leave the prostate and you will die of something else, but with prostate cancer.

Finally, a lot of new drugs are extending the end of life, and we need some drugs – either existing or new agents – that have a curative effect, rather than an end of life effect.

SC: It is important to start with a better understanding of who is at risk from prostate cancer; for individuals and their primary care physicians (GPs) to understand whether or not they’re at higher than average risk, and to be able to then make an informed decision of whether prostate cancer tests like PSA testing are right for them. Risk assessment needs to be a combination of awareness – so that men are aware of the risk factors for prostate cancer and what they should be doing about them, whether they should be acting on that – and also for GPs to understand that, when a man is in front of them, he may have risk factors such as his ethnicity, family history, or age, that put him at higher risk of prostate cancer, and to start a conversation about whether or not a PSA test is the right thing.

A risk tool that would better stratify men into those who may need a test and those who are unlikely to be impacted by aggressive prostate cancer would help both men and their GPs with that conversation. As you go further through the pathway, better diagnostics – a better understanding of a whether a man has prostate cancer or not, what type of prostate cancer it is – would be helpful in understanding whether it needs treatment and then what the most effective treatment is. Finally, when a man has been given a choice of treatments that he is able to access, he should have the full support for all of the side effects that he needs. All of these things are part of one big picture and they can all be solved. Even without any further advances we think that men would have a better deal and better outcomes than they do now if they all received the best available standard of care, notwithstanding any new research that might make treatment or diagnosis even better.

### 3. What are your current research and policy priorities?

SC: At the moment, we have based our policy priorities around those three stages of the prostate cancer journey: the diagnosis, treatment, and support for side effects. Currently, a key policy priority is to improve the diagnosis process that we have at the moment. We know that the PSA test is not ideal, but we also know that the guidelines that exist to help GPs and men decide whether PSA testing is right for them are not being followed consistently, they’re not understood, and we believe that something quite simple could be done to ensure that at least everyone is on the same page with what we have now. We are currently working on getting a consensus across the medial professions in the UK as to best practice for PSA testing as it stands. The consensus will address whether GPs should initiate conversations, who they should be talking to, and whether men should be offered repeat testing – these questions are not all necessarily going to be answered by years of randomized controlled trials. At the moment, there is a lot of inconsistency in what GPs are saying and doing and we need to improve this.

We feel that a small step would just be to get that consistency and that consensus agreed across the UK; for all men to understand their rights to a test if they want one, to be given the right information, and to be managed appropriately by a GP if they do ask for a test. That is the first stage.

The second stage is looking at the availability of treatment across the UK. For example, we know that access to drugs for men with advanced prostate cancer is very different depending on which country they live in. We also know that in parts of the UK, some treatments just aren’t available, such as high dose rate brachytherapy or robotic surgery. We are looking at how we can improve the situation for those men.

In particular, we are working with a coalition of charities within England to work with NHS England, the Department of Health, and pharmaceutical companies, as well as NICE, to see if we can improve the cancer drug commissioning process, we hope to ultimately get affordable, effective drugs to men who need them more quickly.

In terms of supportive side effects, from a policy perspective, we are looking at support for erectile dysfunction. We know that many men who have treatment for prostate cancer will experience erectile dysfunction as a result of it. Their results in terms of sexual function can be improved with early treatment and also the right treatment, but the clinical practice guidelines are out of date. We are working to try to update those treatment guidelines in England. We’ve got guidelines that we have developed with clinical experts that we would like to have adopted. We are also working in coalition with other organisations who are interested in erectile dysfunction services for other disease areas, to see if we have shared areas of improvement that we can work together to achieve.

IF: Prostate Cancer UK at the moment is following a research strategy that has three main priorities. One is to develop a risk assessment tool. The second is to be able to differentially diagnose aggressive versus non-aggressive prostate cancer. The third is developing new treatments for advanced disease.

No strategy stands still, so we are looking at the main aims of the strategy, and to the three priorities above I would add prevention. This does not mean long-term expensive trials or dietary supplements; I think we want to be able to take advantage of some of the research that’s coming out where obesity is being identified as a possible risk marker for prostate cancer. We may be able to collaborate with those working in type II diabetes or cardiovascular disease, to have better effect and help – not just in primary prevention, but actually to help – the secondary outcomes for prostate cancer.

Additionally, I think that we need to be looking at repurposing of existing drugs. We have seen some evidence coming through where drugs that have been used for different cancers could be potentially beneficial in prostate cancer. Repurposing drugs helps drugs to reach the market more cheaply; they have less of the rigorous trials to go through because they have already undergone a lot of safety testing. Our key priorities are the ones that would have the most benefit to the most men.

### 4. Why do we need better risk assessment tools? Could you give a brief outline of some new tools under development?

IF: There are a number of risk assessment tools effectively on the market that have been developed and validated. The problem is that they are not really in routine use. We looked at what was out there, and who was developing them, and we decided to develop a risk assessment tool that was suitable to be delivered through primary care, that was going to be reliable, relatively cheap, and suitable for a multi-ethnic population. So we brought together the experts that had developed those other tools.

I think that the problem is that no matter how complicated the maths or the science behind a risk assessment tool is, it has got to be very easy to use and interpret. We are working with a core group of scientists to help us develop that. The important thing is that there are a number of tools out there that we think would be useful – and we’re following the development of those – but actually what we want is something that is suitable and relatively easy to use.

We see risk assessment as a key priority because it will take some men out of the system who do not need to be tested further or on a regular basis. There will be others who are fired into the system and they have the best treatments available. Then there will be those that do not fall into these categories, so we have been looking at what happens to those men. It should not be a perpetual loop of coming back and getting re-tested with the same old test. It is important to establish what additional tests might be needed to help make the decision of whether you become a red – and have to go and see the urologist or oncologist; or green – and you can go away and not come back for several years. So this is where we are at. It’s a case of honing down and getting something that is actually of practical value.

### 5. Is there a need for better diagnostic tools in prostate cancer?

SC: There absolutely is a need for better diagnostic tools. For a start, being diagnosed with ‘a problem with your prostate’, as the PSA test does and the digital rectal examination does, can lead men on a journey that involves treatment and associated side-effects that they may not need. For some men, detection may not be early enough or treatment effective enough if they have already got advanced disease or go on to develop advanced disease.

We really need to ensure that diagnosis is effective and will lead to men getting the best management for themselves and their disease. Be that, perhaps, active surveillance if they’re considered to be at low risk of developing an aggressive prostate cancer, or more conservative treatment at the right time, and the most effective treatment for that man’s particular cancer.

Diagnosis needs to be improved in two ways. There needs to be a better way of diagnosing that a man actually has a prostate cancer, in an easy way, but also that, prognostically, clinicians can understand whether or not that disease is going to harm that man in his lifetime, and how best to treat it. It is a very key role in the whole prostate cancer journey.

One of the biggest issues is whether screening can be developed. Is there a tool or a test that can be developed in the future that would allow screening for men? That is a big policy question, and it is a big policy challenge. We know that the PSA test is not effective for screening at a population level, but we believe there must be something out there in the future that could help men understand their risk of developing the disease, whether they go on to have further tests, and whether there is a way of diagnosing and detecting aggressive cancers at an early stage, so that we can reduce the number of deaths from prostate cancer.

IF: We urgently need new diagnostic and prognostic tests. I would take it beyond just diagnosis. In prognosis, we are looking to be able to detect, as early as possible, whether a cancer is likely to be aggressive or not. This would allow you to treat the cancer appropriately, and make sure that men know how it will be treated.

It is also important to monitor treatment. We need to be able to follow whether a treatment is working effectively or not. At the moment, it is purely based on PSA. We need to look at other biological markers or genetic markers that will determine whether or not a man is responding to treatment, and if not, then get him off that treatment and onto another treatment sooner. I think, ultimately, what I would like to see is the diagnostic and prognostic tests fitting in with the risk assessment, so they can be done at the same time. Yes, you’re at risk of prostate cancer – or, yes you do have prostate cancer – it is aggressive, and it will respond to this treatment. If we can obtain more knowledge as a package, it will help develop better treatments and we will be able to offer a man a better deal with greater availability of different treatment choices.

### 6. What are the current gaps in care for men with prostate cancer, and what work is currently underway to address these?

SC: There are quite a few gaps in care. For any man with a question about prostate cancer who goes to his GP, it is a bit of a lottery as to whether he will get the information that he should about the risks, about PSA testing and its pros and cons. We know that some GPs feel that they do not have enough information to be able to provide that to a man and support him in that decision. Other GPs will have strong opinions either for or against testing, which may influence their patient – in this case, the patient’s choice would not necessarily be the same as if he was given balanced information and supported to make a decision. Ensuring that the right information reaches GPs and patients is a challenge, as is being able to raise awareness of prostate cancer and the complexities at that pre-diagnosis stage.

The other big gaps are in access to treatment. When a man is diagnosed, he does not necessarily get the treatment that is most appropriate for him. This can be because the first clinician he speaks to does not necessarily give him the full range of options that he could choose, or could be because the clinician may want to prescribe a certain treatment that is not available. Depending on where you live in the UK, you may or may not be able to access certain cancer drugs. Also, things like robotic surgery or high-dose brachytherapy are not available as standard across the country, and these treatments will be important for certain individuals.

The final gap in care is in terms of support. At the moment, treatments do come with side effects. Many of those are unavoidable, but men can be supported – sometimes from before the treatment starts – to reduce the impact of that side effect, be that incontinence or erectile dysfunction, be that the psychological impact of prostate cancer, fatigue, or bowel incontinence. We are told by the health providers that these support services exist, but men are saying that they are not being directed towards them or they are not getting appropriate help at an early enough stage – or any help in some cases. This is a key gap that is easy to close; it is about joining up men with services. In fact, we hope we would ideally save money because if caught early enough then these men might not develop more serious side effects, or impacts of side effects, that need more prolonged treatment.

### 7. Are there any other issues in prostate cancer research and policy that need to be addressed?

IF: I think, no matter how good the science is, we need to make sure there is a cadre of people being able to carry out that science. It is not just in prostate cancer – it’s across medical research in general – we are told that when current clinical researchers retire, there is no one coming through to take their place. We have to make sure that we’re embedding people; not only training them in prostate cancer research, but making sure that the good ones stay in prostate cancer research. Researchers will, quite rightly, move around depending on where the high science is taking place, but we want to bring them into prostate cancer research and keep them there and make sure that they can help deliver on all this great new research that is coming forward.

One thing I would highlight is that we are identifying areas where there is a need to train different disciplines and bring them into prostate cancer. One is pathology – academically trained pathologists – but the other is genetic analysis. A lot of emerging genetic analysis work is reliant on trained mathematicians or statisticians to take part. So we are looking to get these specialists interested in prostate cancer research, and they will be the ones who are analysing these complex data and helping us move forward.

The third thing I would add to that is that we have to make sure that when these great research results come forward, we translate them into patient benefit as soon as possible. We all read scientific papers or press reports on new genes being found for this, that, and the next thing, or new biological markers; it is our role to make sure that the results of such research are then translated into clinical practice as quickly and efficiently as possible.

SC: When we look forward, there are some concerns particularly about the workforce. At the moment, we know that the majority of men with prostate cancer get access to a named nurse specialist. Following some research that we have carried out, we have concerns that there are not enough new nurses to make up for the gaps in the number of nurses we believe will be retiring in the next 10 years. It will be key for health providers in the future to ensure that they have enough specialised nurses, and that those nurses are trained and able to support men.

### 8. What is your future outlook on these issues?

IF: I am hugely optimistic. I think the time is right for us now in research. I think there are some great things coming through and it is for us to capitalise on, and to bring others with us. We will never be able to do this on our own, and this is about other charities, other funders of research joining us, and us being able to identify what the questions are – maybe even how to go about finding the answers to them – and then bringing us all together to make sure that it happens. For us, it is about making sure that whatever great research is going on, it reaches the patient as quickly as possible.

SC: At Prostate Cancer UK, we are developing a new strategy as we speak and we hope to publish it later this year. The strategy will be looking to make prostate cancer a disease that men can live with rather than die from, and to reduce the impact of the disease on men and their families over the next 10 years.

From a policy perspective, there are some key areas for future development. We do need to really invest in research and advances in diagnosis and treatment and prevention, but we also need to use those advances – the advances that are coming through now – and translate that into the best possible care for men now. If we could ensure that every man across the country has the best available care today, we would already see improvements in outcomes. If we can build on that by ensuring that new advances and cost-effective new treatments are able to reach men quickly, then we would see even better outcomes for men. I think this is where we need to be focussing in the future.

### 9. What is your view on the importance of open access publishing for prostate cancer research?

SC: I think open access publishing is important across all of prostate cancer. Obviously, it is vital for researchers who would like to understand what’s going on in the field and to be able to share and learn from their colleagues and peers. However, it is not just people who are researching in the labs who need that information. Charities such as Prostate Cancer UK pride ourselves on our expertise and we have people with research backgrounds who look at the published research and learn from it to inform what we would like to see for men with prostate cancer.

So open access publishing benefits more than just researchers. Clinicians, charities, advocates for men with prostate cancer, all need to see what the latest findings are, what works, what we can learn from each other, and together ensure that we are using that to improve things for men.

IF: I think open access publishing is hugely important, not just for prostate cancer research, but for all research. We have to learn from other disciplines; we have to be able to teach other disciplines or give them information that’s coming through. To get that into the public domain as quickly and easily as possible is absolutely paramount. There is no point in people holding on to information that might help others, so I think that open access publishing is hugely important. We have it written into our terms and conditions that researchers that we fund investigate open access publishing and try to publish open access as much as possible.

### 10. Where can I find out more?

See reference list [1–3].

For further information on prostate cancer and the work of Prostate Cancer UK, please visit www.prostatecanceruk.org

## Additional file

Additional file 1:
**Current challenges in prostate cancer: an interview with Prostate Cancer UK.**
